# Pharmacological Transdifferentiation of Human Nasal Olfactory Stem Cells into Dopaminergic Neurons

**DOI:** 10.1155/2019/2945435

**Published:** 2019-05-19

**Authors:** Audrey Chabrat, Emmanuelle Lacassagne, Rodolphe Billiras, Sophie Landron, Amélie Pontisso-Mahout, Hélène Darville, Alain Dupront, Francis Coge, Esther Schenker, David Piwnica, Emmanuel Nivet, François Féron, Clotilde Mannoury la Cour

**Affiliations:** ^1^Institut de Recherches Servier, Croissy sur Seine 78290, France; ^2^Aix Marseille University, CNRS, Institut Neurophysiopathologie, Marseille, France; ^3^CIC Biothérapies: Inserm CBT-1409, Marseille, France

## Abstract

The discovery of novel drugs for neurodegenerative diseases has been a real challenge over the last decades. The development of patient- and/or disease-specific *in vitro* models represents a powerful strategy for the development and validation of lead candidates in preclinical settings. The implementation of a reliable platform modeling dopaminergic neurons will be an asset in the study of dopamine-associated pathologies such as Parkinson's disease. Disease models based on cell reprogramming strategies, using either human-induced pluripotent stem cells or transcription factor-mediated transdifferentiation, are among the most investigated strategies. However, multipotent adult stem cells remain of high interest to devise direct conversion protocols and establish *in vitro* models that could bypass certain limitations associated with reprogramming strategies. Here, we report the development of a six-step chemically defined protocol that drives the transdifferentiation of human nasal olfactory stem cells into dopaminergic neurons. Morphological changes were progressively accompanied by modifications matching transcript and protein dopaminergic signatures such as LIM homeobox transcription factor 1 alpha (LMX1A), LMX1B, and tyrosine hydroxylase (TH) expression, within 42 days of differentiation. Phenotypic changes were confirmed by the production of dopamine from differentiated neurons. This new strategy paves the way to develop more disease-relevant models by establishing reprogramming-free patient-specific dopaminergic cell models for drug screening and/or target validation for neurodegenerative diseases.

## 1. Introduction

Neurodegenerative disorders such as Alzheimer's disease (AD) and Parkinson's disease (PD) represent complex pathologies for which complete models are difficult to generate. Besides animal models, multiple cell-based models are also being developed, each with their advantages and limitations [1]. Early drug discovery relies mainly on *in vitro* models including primary neuronal cultures, immortalized neuronal-like cell lines, or neurons derived from human-induced pluripotent stem cells (hiPSCs). Although immortalized lines do not closely mimic human phenotypes [2], hiPSC-based neuronal models have been shown to generate human neuron-like cells that can phenocopy disease-specific mechanisms, making these tools useful for compound screening and lead candidate validation among others [3]. However, reprogramming human somatic cells into iPSCs is tedious and remodels the existing somatic epigenetic memory inherent to the patient, with notable effects on aging-associated markers [4, 5]. Consequently, the loss of epigenetic signature in patients during iPSC reprogramming may represent a limitation for certain disease-modeling approaches, especially in the field of neurodegenerative diseases where aging is an important factor. Generating iPSCs may produce off-target integrated mutations, leading to oncogenic consequences. To overcome these drawbacks, preclinical models require more disease-relevant approaches to identify and validate new targets. Recently, direct lineage conversion of somatic cells, such as the transdifferentiation of mouse or human fibroblasts into neurons or neural stem cells, has gained interest in regenerative medicine and also in the development of more disease-relevant models. It has been reported that cell reprogramming *via* transcription factor-mediated transdifferentiation is a gentle process during which aging-associated information is preserved [4]. These studies highlight the potential of patient-specific disease models based on direct differentiation or direct conversion strategies as suitable alternatives to hiPSC-based models. This alternative model is of real interest in pathologies such as PD, in which 90% of cases are not of genetic origin. Thus, the use of cellular models that conserve aging-associated information is necessary for the validation of lead candidates for sporadic forms of PD.

In this context, human adult stem cells represent an interesting model to investigate and are widely used in basic and clinical research to (i) detail intricate developmental mechanisms, (ii) identify pathology-associated biomarkers, (iii) validate new targets and screen molecules, and (iv) implement regenerative medicine [1]. To achieve any of these goals, the appropriate stem cell subtype must be selected. Although adult brain neural stem cells represent the most appropriate starting material from which to derive neuronal models, its inaccessibility often necessitates the use of alternatives. In the domain of brain research, human nasal olfactory mucosa are highly relevant, as they harbor multipotent stem cells [6–8] belonging to the ectomesenchymal stem cell family [9]. Olfactory tissue is derived from the neural crest and thus remains in a state of embryo-like development. The surgical procedure required to obtain nasal stem cells from healthy donors or patients is minimally invasive and performed under local anesthesia [10]. Olfactory ectomesenchymal stem cells (OE-MSCs) have been successfully purified from biopsies and their propensity to differentiate into neuronal cells makes them the model of choice to obtain dopaminergic (DA) neurons [11].

In this study, a pharmacological approach was used to explore direct differentiation/conversion of OE-MSCs from healthy donors. Over the past few years, different approaches have been developed to allow *in vitro* generation of human DA neurons, resulting in more or less effective strategies. Most effective protocols available so far make use of pluripotent stem cells, either of embryonic origin or in a pluripotent state obtained *via* nuclear reprogramming prior to differentiation (hiPSCs). Alternatively, virus-based integrative strategies have been developed to induce cell type-specific genetic changes through transdifferentiation.

In order to develop new and alternative strategies, the present study focused on virus-free protocols using exclusively small molecules [12] to differentiate or transdifferentiate adult human nasal OE-MSCs directly into DA neurons. Several published studies using other cell types [13–19] and well-established differentiation steps to obtain midbrain DA neurons from progenitors [1, 20–22] were considered, and more than 15 combinations of multiple small molecules were tested to obtain an efficient protocol. The resulting DA neurons exhibit morphological features of human neurons and express markers confirming their DA-like status.

## 2. Materials and Methods

### 2.1. Ethical Statement

All OE-MSCs used in this study were obtained from human biopsies from healthy donors, in accordance with the local ethical committee (Comité de Protection des Personnes) of Marseille. All individuals involved in this study provided informed consent in accordance with the Declaration of Helsinki and French laws relating to biomedical research [9, 23].

### 2.2. OE-MSC Culture and Transdifferentiation

Isolated OE-MSCs were cultured in Dulbecco's modified Eagle's medium/nutrient mixture F-12 (DMEM/F-12) supplemented with 10% fetal bovine serum (FBS), 1% penicillin/streptomycin (P/S), 1% GlutaMAX (200 mM; Thermo Fisher Scientific, Waltham, MA, USA), and 1/1000 Plasmocin™ prophylactic (InvivoGen, San Diego, CA, USA), hereafter referred to as complete medium (CM). Transdifferentiation was performed according to the process. After amplification in CM, cells were transferred to flasks precoated with FBS (0.1 mL/cm^2^). For at least 15 days (induction step), cells were grown in freshly prepared medium containing DMEM/F12-neurobasal medium (1 : 1), 1/100 50X B27 (without vitamin A), 1/200 100X N2, 1/1000 2-mercaptoethanol, 1/100 L-glutamine, and P/S. This medium (N2B27) was supplemented with 10 *μ*g/mL fibroblast growth factor 2 (FGF2) and 25 *μ*g/mL activin A and was changed every other day. Transdifferentiation towards DA neurons started on Day 0 (Monday) by switching to N2B27 medium supplemented with 10 mM PD0325901. On Day 1, half of the medium was exchanged for freshly prepared medium identical to the one prepared on Day 0. On Day 2, cells were passaged into wells precoated with poly-L-ornithine (PLO), fibronectin, and laminin, as previously described by Kirkeby et al. [15]. Briefly, 0.2 mL/cm^2^ PLO (15 *μ*g/mL) was added to wells and incubated overnight at 37°C. After removing the PLO solution, wells were washed three times with sterile H_2_O. Fibronectin in phosphate buffered saline (PBS; 0.5 mg/mL) was added and incubated at 4°C for 15-30 min before adding 5 *μ*g/mL laminin immediately before transferring into the prewashed wells (0.2 mL/cm^2^) and incubating overnight at 37°C. Wells were washed once with PBS before plating. N2B27 medium was used without any supplementation for the passage on Day 2. On Day 4, N2B27 medium, supplemented with 0.1 *μ*M human sonic hedgehog (Shh, C24II), 0.1 *μ*g/mL FGF8, and 2 *μ*M CHIR99021, was used. On Days 7 and 9, the medium used on Day 4 was supplemented with 2 *μ*M purmorphamine. On Day 10, the medium contained DMEM/F12-neurobasal medium (1 : 1), 1/50 50X B27 (without vitamin A), 1/100 100X N2, 1/100 P/S, 10 ng/mL brain-derived neurotrophic factor (BDNF), 10 ng/mL glial cell line-derived neurotrophic factor (GDNF), 200 *μ*M ascorbic acid, 0.5 mM N6,2′-O-dibutyryladenosine 3′,5′-cyclic adenosine monophosphate (AMP), 2 *μ*M CHIR99021, 0.01 *μ*g/mL human recombinant neurotrophin-3 (NT-3), 0.5 *μ*M LDN-193189, 0.1 *μ*g/mL human Noggin, and 10 *μ*M SB-431542. After this, only half of the medium was changed every other day (Mondays, Wednesdays, and Fridays) until cells were used for further experiments. Details for product references are available in Table S3.

### 2.3. Immunocytochemistry

All cells were incubated in 4% paraformaldehyde and 4% sucrose in PBS containing calcium and magnesium (PBS Ca^2+^/Mg^2+^) for 15 min. Cells were then washed in PBS Ca^2+^/Mg^2+^ and incubated in 50 mM NH_4_Cl for 20 min. Cells were permeabilized using 0.1% Triton X-100 for 5 min and blocked with 3% bovine serum albumin (BSA) in PBS for 40 min. Cells were incubated overnight at 4°C with the primary antibody in PBS and 3% BSA. Secondary antibodies were diluted in PBS supplemented with BSA (1%) and 4′,6-diamidino-2-phenylindole (DAPI, 1 *μ*g/mL) and added to cells for 45 min after washing with PBS. Finally, before the last PBS wash, cells were incubated for 10 min in CellMask Orange solution (0.5 ng/mL) diluted in PBS. All antibodies used for this study are listed in Table 1.

### 2.4. RNA Extraction and Bioanalysis

Cells were lysed in RLT buffer from RNeasy Micro Kit (Qiagen, Hilden, Germany). Cellular RNA was extracted using QIAcube (Qiagen), according to the manufacturer's protocol. After extraction, RNA was quantified using a NanoDrop 2000 spectrophotometer (Thermo Fisher Scientific). RNA purity was assessed by measuring the ratio of absorbance at 260/280 nm (A260/280 ratio = approximately 2) and 260/230 nm (A260/230 ratio = approximately 2). RNA integrity was assessed *via* microfluidic electrophoresis, using an Agilent RNA screen tape assay on a 4200 Tape Station system (Agilent Technologies, Santa Clara, CA, USA), in accordance with the manufacturer's protocol. An RNA integrity number (RIN) between 7 and 10 indicates high RNA quality.

### 2.5. Retrotranscription and Preamplification

Total RNA (1 *μ*g/50 *μ*L) was reversed-transcribed into cDNA using random primers, deoxyribonucleotide triphosphates (dNTPs), RNAse inhibitor, and multiScribe RT enzyme using a High-Capacity cDNA Reverse Transcription kit (Thermo Fisher Scientific), with the following heating cycles: 25°C for 10 min, 37°C for 2 h, and 85°C for 5 min. Assuming a retrotranscription efficiency of 100% and a final volume of 100 *μ*L, the cDNA concentration was estimated to be 10 ng/*μ*L.

Specific target preamplification of 1.25 *μ*L cDNA was carried out with a mixture of 1.25 *μ*L TaqMan™ Gene Expression Assay (0.2X, Thermo Fisher Scientific, MA, USA) and 2.5 *μ*L TaqMan Preamp Master Mix 2X (Thermo Fisher Scientific), at 95°C for 10 min, followed by 15 PCR cycles, at 95°C for 15 s and 60°C for 4 min. The preamplification product was diluted 1 : 5 in TE buffer (10 mM Tris, 1 mM EDTA, pH 8.0; Teknova, Hollister, CA, USA).

### 2.6. qPCR Analysis

Real-time PCR was performed using Fluidigm 96.96 Dynamic Arrays with BioMark™ HD system (Fluidigm, South San Francisco, CA, USA), according to the manufacturer's protocol. Briefly, 2.25 *μ*L cDNA was combined with 2.5 *μ*L TaqMan Universal PCR Master Mix (2X) (Thermo Fisher Scientific) and 20X GE sample loading reagent (Fluidigm), before loading onto a 96.96 Dynamic Array integrated fluidic circuit. Similarly, 2.5 *μ*L TaqMan assays (Table S2) and assay loading reagent 2X (Fluidigm) were combined before loading onto a 96.96 Dynamic Array integrated fluidic circuit.

### 2.7. Bioinformatic Analysis

To evaluate assay efficacy, Ct values were calculated using the BioMark Real-Time PCR Analysis software (Fluidigm). Ct values between 40 and 24 corresponded to detectable expression levels and values < 24 represented quantifiable expression levels. Ct values were normalized to those of endogenous controls by subtracting average CCCTC-binding factor (CTCF) and TATA-binding protein (TBP) expression levels, using OmicSoft™ software (Qiagen). The most stable reference genes for normalization were identified using a geNorm test on qbase^+^ software (qPCR analysis). A second normalization was performed using a common experimental control sample in the three experiments to correct potential technical qPCR bias. The OmicSoft™ Array viewer version 10.0.1.96 software was used for normalization, and -ΔCt was represented using boxplots according to the median, standard deviation of groups, and heatmaps.

### 2.8. Quantification of Dopamine Synthesis

Cellular dopamine was quantified *via* high-performance liquid chromatography (HPLC). The mobile phase comprised H_2_O, 25 mM citrate monosodium, 1.8 mM decanesulfonate, and 23% *v*/*v* methanol at pH 5.7 (adjusted using concentrated NaOH) and was filtered through a 0.45 *μ*m filter. A Betasil column (Thermo Fisher Scientific; 250 × 2.1 mm, C18, 5 *μ*m) was used and maintained at 35°C using a column heater (Gecko 2000). The flow rate was 0.3 mL/min. For detection, a Coulochem III electrochemical detector, equipped with a cell 5041 and a 13 *μ*m spacer, *E* = +280 mV vs. palladium guard cell *E* = +450 mV, was used.

Standards were prepared for noradrenaline (NA), dopamine (DA), and serotonin (5HT) at 1, 5, 10, and 50 pg, resulting in retention times of 9, 19, and 34 min, respectively. Cells were detached, crushed in 60 *μ*L perchloric acid (0.1 M), and put on ice for 30 min. Samples were centrifuged at 22,000×*g* at 4°C for 30 min. Supernatants (55 *μ*L) were transferred to HPLC tubes (SUN-SRI) in order to inject 50 *μ*L. Data were analyzed using Azur 5.0 software (Datalys, France).

### 2.9. Mass Spectrometry (MS)

Cell lysate pellets were analyzed *via* MS. HClO_4_ was removed from the pellets, which were resuspended in 40 *μ*L deoxycholate (2% in 50 mM ammonium bicarbonate). After vortexing, samples were incubated for 5 min at 95°C. After centrifugation for 2 s, 5 *μ*L was used for protein quantification. To each sample, 10 *μ*L 1 mg/mL trypsin (Trypsin type IX-S, Sigma-Aldrich, St. Louis, MO, USA) was added, and the samples were vortexed, before digesting in an ultrasound bath (47000 Hz, 130 W, Branson 1200) for 30 s. Digestion was stopped by adding 1 *μ*L formic acid (HCOOH, Fluka 26.5 M). The digest was vortexed, centrifuged for 30 min at 22,000×*g*, and the supernatant was transferred to a tube adapted for MS analysis. Analyses were performed using an LC-MS-8060 instrument (Shimadzu, Kyoto, Japan). The six most abundant ions were selected for tandem MS (MS/MS) detection. Separation was performed using a XBridge peptide BEH C18 column 130 Å, 3.5 *μ*m, 2.1 mm × 150 mm (Waters Corporation, Milford, MA, USA). The mobile phases used comprised 0.1% formic acid in acetonitrile.

### 2.10. Western Blot

Cells were washed twice in ice-cold PBS and lysed in radio-immunoprecipitation buffer (RIPA buffer, Sigma-Aldrich) supplemented with 1/100 protease inhibitor (Calbiochem, San Diego, CA, USA) and 1/100 phosphatase inhibitor (Calbiochem). Cells were harvested in 2 mL aliquots, transferred to Precellys tubes for homogenization, centrifuged at 34×*g* for 30 s, and placed on ice for 30 s. This protocol was repeated eight times. Cells were kept on ice for 30 min. Cell debris was separated from proteins *via* centrifugation at 19,400×*g* for 10 min at 4°C. Five microliters of the supernatant was used for protein quantification using a Pierce bicinchoninic acid (BCA) protein assay kit (Thermo Fisher Scientific) and a Victor instrument (Perkin Elmer, Waltham, MA, USA).

Samples with a total protein concentration of 15 *μ*g (or 25 *μ*g, were prepared in 4X Laemmli buffer (Bio-Rad, Hercules, CA, USA). Samples were heated at 95°C for 5 min and loaded onto NuPAGE™ Novex 4-12% Bis-Tris Gels (15 wells, Thermo Fisher Scientific). Prestained standards (SeeBlue™ Plus 2 and MagicMark™ XP, Thermo Fisher Scientific) were included in the outer lanes. Transfers were performed in NuPAGE Transfer Buffer 1X, after which nitrocellulose membranes (0.2 *μ*m pore size, Thermo Fisher Scientific) were washed once with washing buffer 1X Tris-buffered saline (Bio-Rad) + 0.05% *v*/*v*Tween-20 (Bio-Rad), TBST, and transferred in blocking buffer (TBST + 5% BSA) for 1 h. Membranes were incubated overnight at 4°C with the primary antibody (see Table 1 for the concentration) in TBST + 5% BSA. After three 10 min washes with TBST, membranes were incubated with the secondary antibody for 1 h before washing three times in TBST. Membranes were then visualized *via* enhanced chemiluminescence (ECL; GE Healthcare, Chicago, IL, USA). Chemiluminescence was measured using ChemiDoc following signal apparition for 5 to 10 min. In order to detect actin on the same membranes, stripping was performed by incubating membranes in Restore Western Blot Stripping Buffer (Thermo Fisher Scientific) for 15 min at room temperature without shaking. Membranes were first incubated in blocking buffer and then in an actin-horseradish peroxidase (HRP) antibody-containing solution.

### 2.11. Microscopes

For immunofluorescence, cells were plated in 96-well plates (CellCarrier-96, PerkinElmer), imaged using the PerkinElmer Opera Phenix™ High-Content Screening System and processed using Harmony software (PerkinElmer). Brightfield pictures were acquired using an Olympus CKX31 microscope (Olympus, Tokyo, Japan) equipped with a digital camera.

## 3. Results

### 3.1. Six-Step Differentiation Protocol

**Figure 1:**
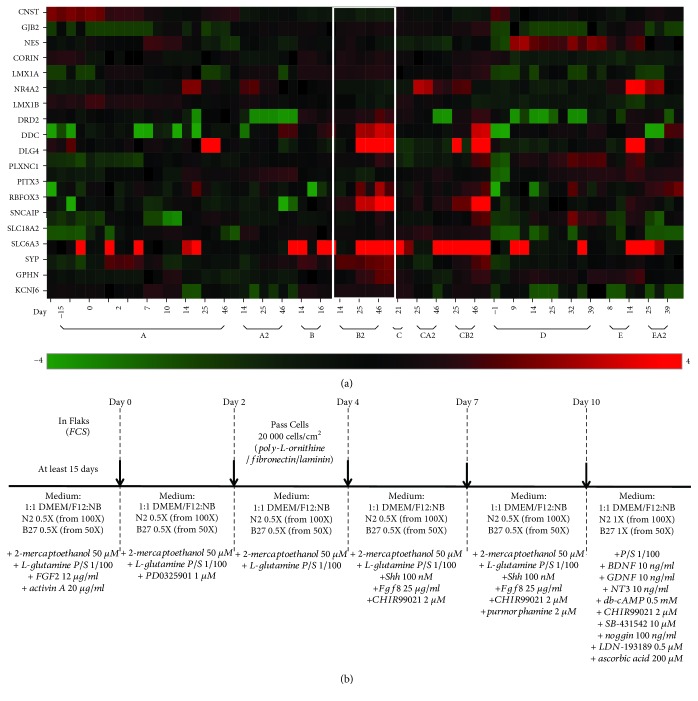
Identification of the most efficient protocol according to gene expression. (a) Heatmap representation of 20 neuron- and/or dopamine-specific genes (*KCNJ6*, *GPHN*, *SYP*, *SLC6A3*, *SLC18A2*, *SCNCAI*P, *RBFOX3*, *PITX3*, *PLXNC1*, *DLG4*, *DDC*, *DRD2*, *LMX1B*, *EN1*, *NR4A2*, *LMX1A*, *CORIN*, *NES*, *GJB2*, and *CNST*) for 10 different protocols, labeled A, A2, B, B2, C, CA2, CB2, D, E, and EA2. Green: underexpressed genes; red: overexpressed genes. Time point labels correspond to Day -15 = untreated stem cells; Day 0 = start of cell differentiation, after an induction period of at least 15 days for protocols A, A2, B, B2, C, CA2, and CB2; durations of differentiation periods are indicated (Day 2 to Day 46). Protocol B2, framed in white, induced the most important overexpression of dopaminergic (DA) neuron-specific transcripts. (b) Schematic representation of the six step-based protocol B2 showing the basic medium (normal font), small molecules (italics), and the coatings with fetal calf serum (FCS), until Day 2, and poly-L-ornithine/fibronectin/laminin from Day 2.

In order to devise a protocol allowing the direct conversion of OE-MSCs into DA neurons, the following parameters were adjusted in each protocol tested: (1) combinations of small molecules used to modulate various signaling pathways [12], (2) concentrations of each tested molecule, and (3) duration of treatment with different combinations of molecules, in a stepwise manner (Table S1). The first two inclusion/exclusion criteria were based on the capacity of OE-MSCs to survive and to change their overall aspect from a fibroblast-like morphology (bipolar with elongated shape) to a neuronal-like morphology (branched cells with round and bright soma) when exposed to a defined protocol. Of the 15 protocols tested, 10 fulfilled these criteria. A gene analysis was performed on cells cultured using the 10 selected protocols to assess whether differentiated cells acquired a gene signature resembling that of DA neurons (Figure 1(a)). To this end, cells were harvested at critical time points in each protocol, corresponding to changes in molecule combinations applied to the culture undergoing differentiation. For each time point, expression levels were evaluated for a preselected panel of 20 genes that determine a DA-like neuronal signature ([1, 20–22]; Table S2). Among the 10 differentiation protocols, one (hereafter referred to as protocol B2) proved to be the most efficient procedure to induce changes in the gene expression profile of OE-MSCs and resulted in a DA-like signature, as revealed *via* heatmap analysis (Figure 1(a); [1, 20–22]). Protocol B2 (Figure 1(b)) includes a 15-day induction phase in DMEM/F12-neurobasal medium (1 : 1), B27 (without vitamin A), N2, 2-mercaptoethanol, L-glutamine, and antibiotics (N2B27 medium), supplemented with FGF2 and activin A. These two molecules were previously shown to synergistically promote early neural differentiation [24]. FGF2 is known to act on cell proliferation [25] and activin A induces neuronal differentiation [26]. The induction phase, common to seven of the 10 tested protocols, did not result in major changes in the expression of selected genes (Figure 1(a)). However, without this induction phase preceding subsequent differentiation, no efficient direct conversion was observed, as demonstrated by the results obtained with protocols D, E, and EA2 (Figure 1(a)). This suggests that pretreatment with FGF2 and activin A, along with the use of N2B27 medium, is necessary to trigger a phenotypic switch in OE-MSCs. The induction phase was followed by four successive differentiation conditions, over a 10-day period. Direct conversion to DA neurons was initiated on Day 0 post induction by adding the MEK/ERK pathway inhibitor PD0325901 for 2 days to the N2B27 medium. This step halted proliferation triggered by the induction medium and redirected the cellular machinery towards differentiation. On Day 2 post induction, cells were plated in wells precoated with PLO, fibronectin, and laminin to provide a substrate known to support differentiation into DA neurons [15]. At Day 4 post induction, human recombinant SHH, FGF8 (STEMCELL Technologies, Vancouver, Canada), and the GSK3 inhibitor CHIR99021 (Abcam, USA) were added to N2B27 medium to activate the WNT pathway, as well as model key events occurring at the isthmic organizer level for midbrain formation. At Day 7, purmorphamine, another SHH agonist, was added to the molecular cocktail for an additional 3-day period. Cells were analyzed at the end of each treatment (Days 2, 4, 7, and 10 post induction). At all time points, changes in the expression profile for the 20 preselected genes remained minor, though an increase in the expression of certain genes (*SLC6A3*, *DDC*, and *NR4A2*) was observed, indicative of a *de novo* phenotypic status more prone to switch to a DA neuron-like profile. At this point, different culture conditions were tested to identify a procedure able to promote the conversion of OE-MSCs into a DA-like neuronal phenotype. The most efficient cocktail identified comprised BDNF, GDNF, ascorbic acid, N6,2′-O-dibutyryladenosine 3′,5′-cyclic AMP, CHIR99021, NT3, LDN-193189 (bone morphogenetic protein (BMP) pathway inhibitor), Noggin, and SB-431542 (activin/BMP/transforming growth factor- (TGF-) *β* inhibitor). Upon addition of this cocktail on Day 25 (15 days induction + 10 days post induction), a dramatic switch in the transcriptomic profile of the selected genes was observed (Figure 1(a)). Cells acquired a DA-like neuron gene signature, with rapidly increasing gene expression levels for most of the genes selected (Figure 1(a)). Analysis at Day 46 indicated that converted cells maintained a DA-like neuronal gene signature over time.

Protocol CB2, which was the most similar to protocol B2, induced similar changes. Protocol CB2 included purmorphamine treatment for 14 days instead of 3 days in protocol B2 (Table S1). Most experiments presented in this study were therefore performed using protocol B2. In order to assess reproducibility of the direct conversion protocol, all subsequent experiments were performed using OE-MSCs derived from three healthy donors.

In order to test whether this direct conversion protocol could be successfully applied to other human somatic cells, the efficacy of this protocol was also tested on human fibroblasts. The use of this newly described conversion procedure showed interesting morphological and gene expression changes during the first phases of the procedure (Figure S1). However, contrary to what has been observed with OE-MSCs previously, skin fibroblasts were not able to survive during the whole procedure, suggesting that this protocol could not be applied for the pharmacological conversion of this cell type.

### 3.2. Differentiation Phases Were Associated with Morphological Transformations

**Figure 2:**
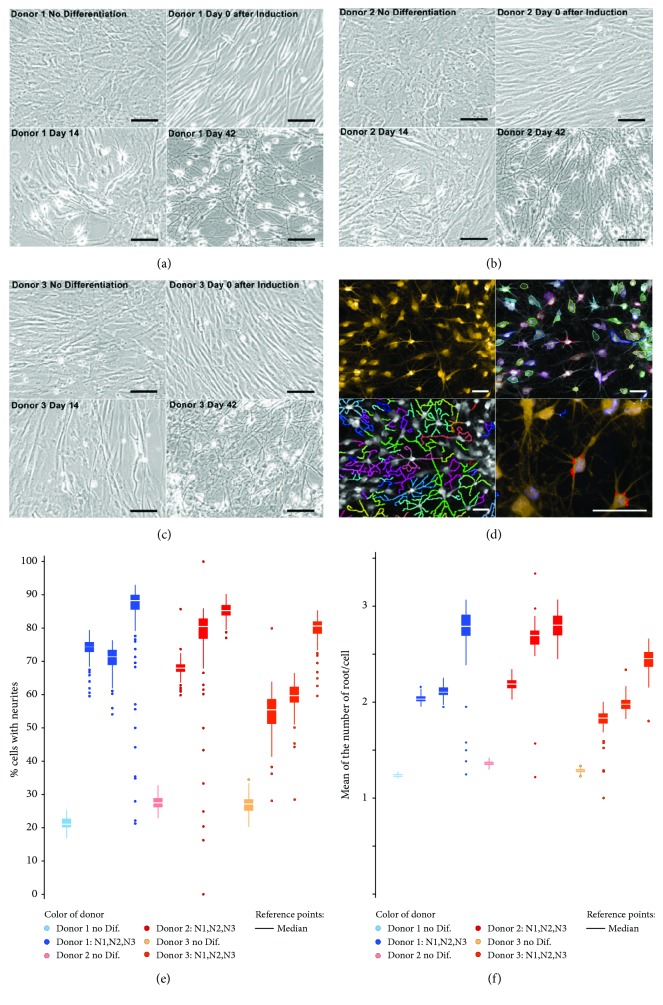
Morphological changes associated with differentiation sequences. (a–c) Brightfield pictures of olfactory ectomesenchymal stem cells (OE-MSCs), from donor 1 (a), donor 2 (b), and donor 3 (c), at four time points corresponding to key differentiation sequences. Scale bar: 100 *μ*m. (d) Top left, OE-MSCs at Day 42, after CellMask Orange staining. Using Harmony software (PerkinElmer), cell bodies (top right) and neurites (bottom left) were delineated. Bottom right, neuritic roots are highlighted in red. Scale bar: 50 *μ*m. (e) Percentage of neurite-presenting cells from the three donors before differentiation and at Day 42. Triplicate experiments (N1, N2, and N3) displayed similar results. (f) Mean number of neuritic roots per cell.

Untreated OE-MSCs display typical mesenchymal stem cell morphology with a limited arborization, as shown in Figures 2(a)–2(c) for the three different donors. During the induction phase, limited morphological changes were noticed and neuritis developed predominantly after changing to the maturation medium on Day 10 (Figures 2(a)–2(c)). Subsequently, the undifferentiated OE-MSCs were directly cultured in maturation medium without going through an induction phase. However, no relevant change was observed (data not shown), thereby demonstrating the importance of the induction phase.

In order to quantify morphological changes, roots with multiple filopodia were counted using a high-content analysis system. Images acquired using an Opera Phenix instrument after CellMask Orange staining (Figure 2(d), top left image) were analyzed using the Harmony software for neurite counting (Figures 2(e) and 2(f)). Comparison between undifferentiated and differentiated OE-MSCs (Day 42) indicated increased hierarchical arborization in differentiated OE-MSCs. Changes in cell body shape and neurite growth agree with preliminary electrophysiological data indicating reduced kappa currents (data not shown). Moreover, modulated gene expression of two types of connexins, *CNST* and *GJB2*, was observed when analyzing the transcriptome of OE-MSCs undergoing differentiation. Underexpression of *CNST* and overexpression of *GJB2* were observed (Figure S2), suggesting a link between cell morphology changes and gap junction modifications [27]. After this morphological analysis, transcript and protein variations were assessed during cell differentiation.

### 3.3. Differentiation Phases Were Associated with Transcript Variations

A number of morphogens and transcription factors implicated in the development of midbrain DA neurons were reported [22]. A selection of 27 genes was used (Table S2) for transcriptomic analysis at critical time points, in order to assess the status of conversion of OE-MSCs into DA neurons.

**Figure 3:**
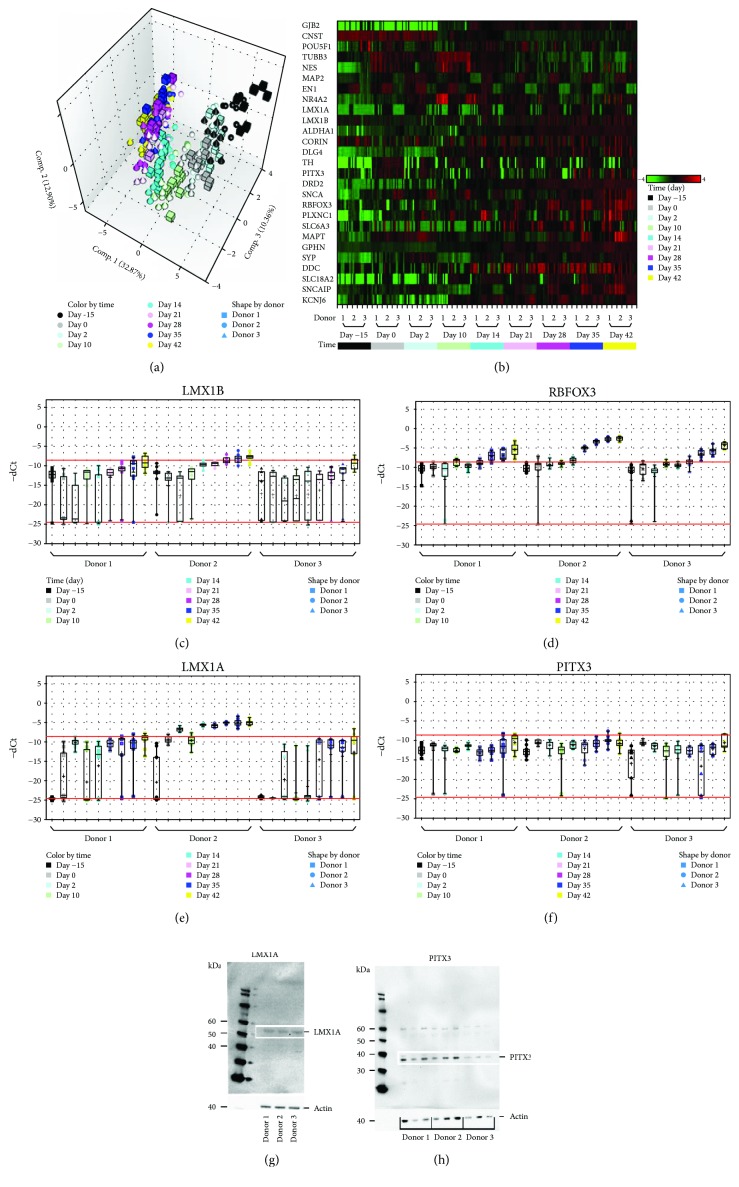
Time-dependent expression of dopaminergic (DA) neuron-specific transcripts and proteins. (a) Principal component analysis (PCA) representation, based on the expression of 27 target genes, indicated a strong correlation between the three donors at various time points: Day -15, Day 0, Day 2, Day 10, Day 14, Day 21, Day 28, Day 35, and Day 42. (b) Heatmap of the 27 target genes. Some genes remained underexpressed (green) along the entire procedure while expression of others increased, resulting in overexpression (red) at the end of the differentiation sequences. (c–f) Time-dependent increase in transcript expression for four major DA neuron-associated genes: *LMX1B* (c), *RBFOX3* (d), *LMX1A* (e), and *PITX3* (f). Bottom and top red lines indicate the thresholds for detection (undetectable below the bottom red line) and quantification, respectively. Between the two red lines, the gene is detected but not precisely quantified as the Ct signal is below the limit of quantification. Above the top red line, the gene is correctly expressed and quantified. (g, h) Protein identification *via* western blotting: at Day 42, OE-MSCs from the three donors produce LMX1A (g) and PITX3 (h).

Principal component analysis (PCA, Figure 3(a)) revealed similar expression patterns for all genes, when comparing cells from the three donors. Overall, three clusters were obtained: cluster 1, untreated cells until differentiation Day 2; cluster 2, cells from differentiation Day 10 to Day 21; and cluster 3, from differentiation Day 28 to Day 42. The three clusters correspond to the three major sequences of the differentiation protocol. Sequence 1 (untreated cells to differentiation Day 2) is associated with proliferation during the induction phase and mimics processes during the isthmic organizer phase. Sequence 2 (differentiation Day 10 to Day 21) marks the beginning of maturation while sequence 3 (differentiation Day 28 to Day 42), which is associated with maturation maintenance and neuronal networking, displays clear expression of all major specific DA neuronal markers. The similarity between donors at the transcriptional level reported above is illustrated in Figure 3(b), and the expression of four important and specific markers of DA neurons (*LMX1B*, *RBFOX3*, *LMX1A*, and *PITX3*) is shown in Figures 3(c)–3(f). A genotypic shift was observed with an increase in the expression of (i) *RBFOX3*, indicating a commitment to neuronal lineage, (ii) *LMX1A* and *LMX1B*, evidencing a step towards DA differentiation and maturation [21, 28, 29], and (iii) *PITX3*, a recognized marker of mature DA neurons. As shown in Figures 3(g) and 3(h), these transcriptional changes were confirmed at the protein level with the detection of LIM homeobox transcription factor alpha (LMX1A) and pituitary homeobox protein 3 (PITX3; approximately 50 kDa and 35 kDa, respectively) in all three OE-MSC cell lines. Among the 27 assessed genes, overexpression of *ASCL1*, *SLC18A2*, *GPHN*, and *DLG4* was also observed during the whole differentiation phase (Figure S3).

### 3.4. Protein Analysis of Specific DA Markers

**Figure 4:**
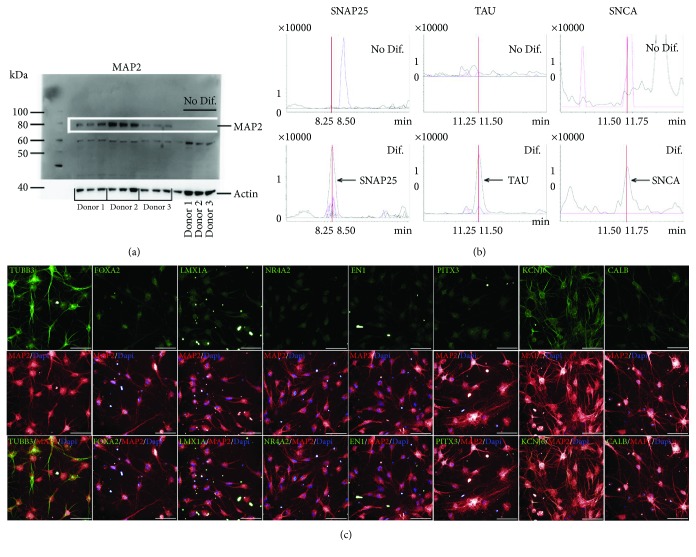
Expression of dopaminergic neuron-specific proteins. (a) Western blot of microtubule-associated protein 2 (MAP2) expression performed in triplicates for all cell lines, at Day -15 and Day 42. (b) Differential detection of synaptosome-associated protein 25 (SNAP25), microtubule-associated protein tau (MAPT), and alpha synuclein (SNCA) in undifferentiated and differentiated OE-MSCs *via* mass spectrometry. As shown here, these three proteins were not detected in undifferentiated cells but were produced by differentiated cells on Day 45. (c) Immunostaining of differentiated OE-MSCs (Day 42) with antibodies raised against tubulin beta 3 (TUBB3), forkhead box protein A2 (FOXA2), LIM homeobox transcription factor 1 alpha (LMX1A), nuclear receptor subfamily 4 group A member 2 (NR4A2), engrailed homeobox 1 (EN1), pituitary homeobox protein 3 (PITX3), potassium voltage-gated channel subfamily J member 6 (KCNJ6), calbindin (CALB, green), and MAP2 (red).

In order to further characterize this new model of cell transdifferentiation into DA neurons, specific protein markers were assessed. Expression of microtubule-associated protein 2 (MAP2), a well-known specific neuronal marker, was investigated *via* western blot. MAP2 was observed at the expected apparent molecular weight from 75 to 80 kDa, corresponding to its C/D isoforms (Figure 4(a)). The experiment was performed in triplicate with cells from three different donors. Similar results were obtained for all donors at Day 42. In comparison, no MAP2 protein was detected in undifferentiated OE-MSCs (Day -15, before induction), a result indicating a shift in protein expression and neuronal maturation.

Using MS, we also compared the expression of synaptosome-associated protein 25 (SNAP25), microtubule-associated protein tau (MAPT), and SNCA in differentiated and undifferentiated OE-MSCs (Figure 4(b)). Again, a phenotypic shift was observed for the three tested proteins. Alpha synuclein (SNCA) and SNAP25 were detected in differentiated cells but not in undifferentiated cells. As reported in Figure 4(b), the neuronal mature form of MAPT was only expressed in differentiated cells (Day 45), indicating true maturation towards DA neurons. Furthermore, multiple immunostaining analyses for the detection of tubulin beta 3 (TUBB3), MAP2, forkhead box protein A2 (FOXA2), LMX1A, nuclear receptor subfamily 4 group A member 2 (NR4A2), engrailed homeobox 1 (EN1), PITX3, potassium voltage-gated channel subfamily J member 6 (KCNJ6), and calbindin (CALB) confirmed differentiation and maturation (Figure 4(c)).

### 3.5. Dopamine Synthesis by Differentiated OE-MSCs

**Figure 5:**
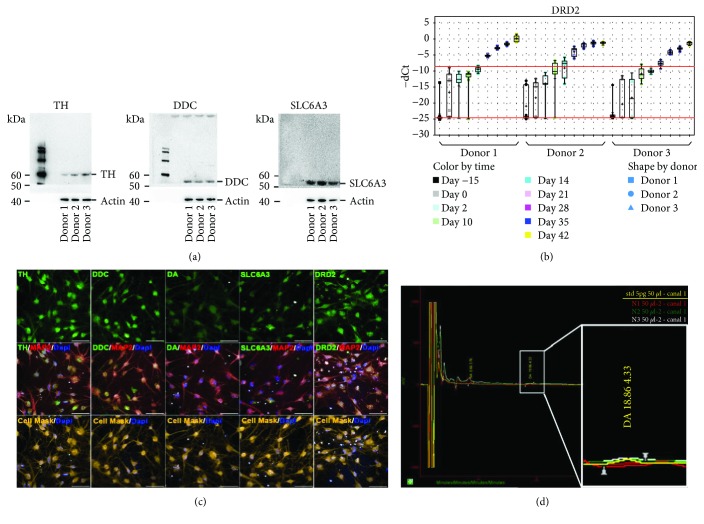
Dopamine synthesis by differentiated OE-MSCs. (a) Western blot for tyrosine hydroxylase (TH), dopa decarboxylase (DDC), and solute carrier family 6 member 3 (SLC6A3). (b) Time-dependent expression of dopamine receptor D2 (DRD2), from Day -15 (no treatment) to differentiation Day 42. (c) Immunostaining at Day 42 with antibodies raised against TH, DDC, dopamine, SLC6A3, and DRD2. (d) Dopamine (DA) was quantified in cell lysates *via* high-performance liquid chromatography (HPLC).

To further characterize the level of maturity of the differentiated cells, dopamine synthesis was evaluated. Western blot analyses were performed to determine the expression of molecules involved in dopamine synthesis and transport. The expression of tyrosine hydroxylase (TH), dopa decarboxylase (DDC), and solute carrier family 6 member 3 (SLC6A3) at Day 45 in all three OE-MSC donor lines is shown in Figure 5(a). As expected, TH, DDC, and SLC6A3 revealed apparent molecular weights of 60, 50, and 55 kDa, respectively. Notably, the SLC6A3 band corresponded to the glycosylated form of phosphorylated SLC6A3. The increase in dopamine receptor D2 (DRD2) gene expression throughout the different phases of differentiation is shown in Figure 5(b). Immunostaining at Day 42 confirmed the presence of molecules involved in dopamine synthesis and also the expression of the dopamine transporter and receptors TH, DDC, and DRD2 (Figure 5(c)).

The total amounts of dopamine measured in samples with three different cell seeding densities (1 × 10^5^, 1.5 × 10^5^, and 2 × 10^5^ cells per well) were 0.36 pg, 4.86 pg, and 10.3 pg, respectively. This indicates a nonlinear increase in dopamine synthesis, which may be due to an interplay between cells and a threshold effect at low cell density. As expected, endogenous dopamine synthesis was enhanced when L-DOPA (50 *μ*M) was added to the medium for 24 h. Enhanced dopamine production was observed in all three cell lines investigated (data not shown).

## 4. Discussion

In view of the need for new disease-relevant *in vitro* models to support drug discovery programs in neurodegeneration, we developed a novel *in vitro* model of DA-like neurons derived from human nasal olfactory stem cells. For this purpose, we validated a transdifferentiation protocol allowing the pharmacological conversion of OE-MSCs through six phases, with specific combinations of signaling pathway modulators.

The final protocol leads to progressive changes in both transcriptomic and protein signatures, with expression of most typical markers of DA neurons. The synthesis of dopamine in differentiated cells confirmed the switch from OE-MSCs towards a DA phenotype upon exposure to a specific cocktail of small molecules. This change of phenotype, from undifferentiated OE-MSCs to differentiated neurons, was also supported by an important decrease in serotonin expression, which was observed after cell differentiation.

This proof-of-concept study demonstrates that human nasal OE-MSCs represent prime stem cell material for the development of new alternative strategies for the *in vitro* production of human DA neurons. Although both transdifferentiated and hiPSC-derived neurons have the ability to recapitulate the pathological features of a patient, only transdifferentiated cells retain certain patient-specific signatures associated with aging, including epigenetic information [4, 5, 30]. This is of utmost importance when considering the establishment of an *in vitro* stem cell-based model to study the molecular processes of neurodegenerative diseases such as PD and AD, in which epigenetic markers of aging may play a primordial role in pathophysiology. Therefore, pharmacological transdifferentiation of patient-specific OE-MSCs into DA neurons may represent a reliable approach to implement scalable disease-modeling platforms suitable for both target validation and compound characterization. This approach is complementary to other relevant models, such as DA neurons obtained *via* transcription factor-mediated transdifferentiation or hiPSC-based differentiation, and could be performed for the discovery of new biomarkers or efficient therapeutic molecules that otherwise may be missed with other strategies because of their respective limitations. To further this study, the epigenetic features of differentiated OE-MSCs obtained with our protocol should be assessed and compared to those of OE-MSCs from the same donor but which underwent a reprogramming step.

The reproducible phenotype obtained with OE-MSCs from all three different donors confirms the robustness and reproducibility of our new established protocol. Analysis of OE-MSCs performed prior to applying the different protocols revealed a transcriptomic profile with very low or no expression of most of the preselected genes. This finding confirms previous reports on the nonneuronal phenotype of OE-MSCs [9] and more importantly reveals that OE-MSCs are not a stem cell subtype already engaged in a DA lineage. Remarkably, we systematically observed the expression of genes and/or proteins known to be key in defining a DA phenotype in cells that underwent differentiation, suggesting the presence of DA neurons inside the differentiated culture. For example, LMX1A and LMX1B are two necessary transcription factors for DA neuronal differentiation, which is known to follow three important steps, each of which is defined by a pool of transcription factors among which LMX1A and LMX1B are always expressed [28, 31]. Thus, developing a cellular model able to express both of these factors is an important feature supporting the shift to a DA phenotype. Importantly, we also succeeded in inducing the expression of markers specific to cellular subtypes of DA neurons. Indeed, midbrain DA neurons are mostly composed of two groups of DA neurons: those localized in the substantia nigra pars compacta (SNpc; [32, 33]) and those present in the ventral tegmental area (VTA; [34]). In our cellular model of transdifferentiated OE-MSCs, we enhanced the expression of KCNJ6 and PLXNC1, which are specific markers of SNpc and VTA neurons, respectively [21]. With regard to PD, SNpc neurons degenerate first [35, 36]. Thus, being able to use a model encompassing both subcellular populations of DA neurons is of real interest to (i) better understand differences in molecular processes underlying neuronal degeneration, (ii) find new biomarkers, and (iii) validate novel therapeutic targets involved in cellular degeneration. In addition to these two markers, our protocol also induced the expression of achaete-scute homolog 1 (ASCL1), a transcription factor commonly used in DA differentiation protocols and which is often overexpressed *via* treatment with viral vectors. Increased expression of *SLC18A2*, *GPHN*, and *DLG4*, usually concomitant with the establishment of a neural network, was also observed. Our protocol also induced expression of FOXA2, NR4A2, EN1, and PITX3, which are specific markers of the maturation stage of DA neurons [22]. However, transcription factors are not the only regulators of DA neuronal differentiation, as several microRNAs (miRNAs) have also been described as major regulators of this process and represent interesting markers to investigate in our new model [37].

Besides these dramatic changes in genetic and protein profiles, dopamine production was detected in OE-MSC-derived cells, as early as 45 days after the start of differentiation. This indicates that OE-MSC-derived DA neurons show some functionality, at least in their capacity to synthesize dopamine and to respond to a chemical stimulus such as L-DOPA. Moreover, the functional maturation of these differentiated cells was confirmed *via* calcium imaging experiments (data not shown). Although only approximately 1% of cells showed calcium activity, these results seem to indicate that a subset of cells was functionally mature at the time of analysis (Day 70 after the start of differentiation). The low density of mature cells may also explain the relatively low levels of dopamine detected in cultures. However, considering that our results revealed the expression of DA neuronal markers in most differentiated cells, a finding that was confirmed by unambiguous profiles at the gene expression level for the whole culture, this probably reflects the presence of DA neurons at different stages of maturity within the culture rather than a heterogeneous and inefficient conversion process. For future applications, it may therefore be necessary to promote cell maturation by increasing the time in culture and developing coculture with astrocytes and glutamatergic or gamma-aminobutyric acid- (GABA-) ergic neurons. In this regard, a recent paper reported that hiPSC-derived DA neurons required 95 days in culture prior to displaying fully mature functions such as action potentials [38].

In addition to their application as disease-relevant *in vitro* models, OE-MSCs may also be used for autologous as well as allogenic transplantation. Interestingly, they can even cross various metabolic barriers [23, 39] and therefore be injected in the cerebrospinal fluid or the blood circulation. Promising results were obtained with various modes of grafting, including animal models of paraplegia [40, 41], cochlear damage [42, 43], and amnesia [23]. It has also been shown that grafted human OE-MSCs generate DA cells and reduce behavioral asymmetry induced by ablation of the DA neurons in a rat model of PD [11, 44]. These studies demonstrate the ability of OE-MSCs to integrate into a microenvironment and become fully mature and functional endogenous components. Thus, in the case of PD, the model described in our study is relevant for regenerative medicine. Transplanted differentiated OE-MSCs may integrate into their nearby environment and mature fully, thus establishing a functional neuronal network and compensating for the loss of endogenous DA neurons.

Transdifferentiation or direct lineage reprogramming holds great potential to generate specific subsets of patient-specific neuronal cells that could be used to implement disease-relevant *in vitro* platforms to (i) study the molecular features of various human diseases, (ii) identify targets, and (iii) screen potential therapeutic drugs [45]. Up to now, the concept of transdifferentiation for the generation of DA neurons has been mainly conceptualized as the direct conversion of somatic identity to another lineage by nuclear reprogramming, mediated by the introduction of lineage-specific transcription factors. After the first report on transdifferentiation by Caiazzo and collaborators [46] that demonstrated the direct conversion of fibroblasts to myoblasts *via* MyoD overexpression, several groups reported the direct lineage conversion of human fibroblasts to DA neurons [19, 47]. Nevertheless, and contrary to that in the present study, all currently available transdifferentiation protocols for the *in vitro* generation of DA neurons make use of the nuclear delivery of DA neuron-specific transcription factors, along with culture conditions supporting conversion to DA neurons. Overall, pharmacological reprogramming of OE-MSCs into DA neurons could represent a major advantage over current transdifferentiation methods, as it does not require the use of an integrative approach to impose a genetic program that may prevent undesired effects associated with such technologies.

## 5. Conclusions

In conclusion, we established a new and alternative method for the *in vitro* generation of DA neuron-like cells from OE-MSCs without requiring the need to go through a pluripotent ground state prior to differentiation, or the use of transcription factor delivery *via* viral approaches. This method represents a significant step forward in the development of a method for the conversion of nonneuronal adult human stem cells to DA neurons, and a credible alternative to the use of hiPSC-derived cells. This strategy offers the possibility to implement patient-specific disease modeling platforms as OE-MSCs can be harvested from PD patients, and may offer opportunities to study disease-specific mechanisms in aged cells such as aggregation of misfolded and fibrillar forms of SNCA [47]. Ultimately, this approach may also represent a useful strategy for implementing new drug discovery tools for the identification of new targets and/or therapeutic molecules for neurodegenerative disorders.

## Figures and Tables

**Table 1:** Antibodies used for immunostaining and western blot.

	Antibodies	Species	Concentration	Origin	Catalog number
Primary antibodies	*β*III-tubulin	Rabbit	IF: 1/1000	Covance	PRB-435P
	Calbindin	Rabbit	IF: 1/200	Abcam	ab25085
	Dopamine	Rabbit	IF: 1/500	Abcam	ab6427
	Dopa decarboxylase	Rabbit	WB: 1/1000 IF: 1/200	Abcam	ab3905
	Dopamine D2 receptor	Goat	IF: 1/200	Abcam	ab30743
	Dopamine transporter	Rat	WB: 1/1000 IF: 1/200	Millipore	MAB369
	Engrailed 1	Rabbit	IF: 1/25	Abcam	ab70993
	Foxa2/HNF3b	Rabbit	WB: 1/1000 IF: 1/400	Cell Signaling	8186
	Girk2	Goat	IF: 1/100	Abcam	ab65096
	Lmx1a	Rabbit	WB: 1/1000 IF: 1/200	Millipore	AB10533
	MAP2	Chicken	WB: 1/10000 IF: 1/5000	Abcam	ab5392
	Nurr1	Mouse	IF: 1/300	Abcam	ab41917
	Pitx3	Rabbit	WB: 1/250 IF: 1/200	Abcam	ab30734
	TH	Rabbit	WB: 1/500	Millipore	AB152
	TH	Rabbit	IF: 1/500	Pel-Freez	P40101-150
	Actin-HRP	Mouse	WB: 1/10000	Proteintech	HRP-60008

Secondary antibodies	Rabbit-HRP	Goat	WB: 1/6000	Cell Signaling	7074
	Chicken-HRP	Goat	WB: 1/6000	Abcam	ab97135
	Rat-HRP	Goat	WB: 1/5000	Millipore	AP136P
	Anti-rabbit-Alexa 488	Goat	IF: 1/1000	Thermo Fisher	A11070
	Anti-goat-Alexa 488	Donkey	IF: 1/1000	Thermo Fisher	A11055
	Anti-mouse-Alexa 488	Goat	IF: 1/1000	Invitrogen	A11029
	Anti-rat-Alexa 488	Goat	IF: 1/1000	Thermo Fisher	A11006
	Anti-chicken-Alexa 647	Goat	IF: 1/1000	Thermo Fisher	A21449
	HCS CellMask Orange		IF: 1/20000	Thermo Fisher	H32713
	DAPI		IF: 1/10000	Sigma Aldrich	D8417

WB: western blot; IF: immunofluorescence; HCS: high-content screening.

## Data Availability

The data used to support the findings of this study are available from the corresponding authors upon request.

## Supplementary Materials

Supplementary Materials Figure S1: comparative study—stem cells versus fibroblasts—of 4 culture protocols. Figure S2: time-dependent expression of connexin 26 and consortin during OE-MSC differentiation. Figure S3: time-dependent expression of 4 dopaminergic markers during OE-MSC differentiation. Table S1: details of the 10 protocols used to differentiate OE-MSCs into dopaminergic neurons. Table S2: list of the 27 genes analyzed. Table S3: list of reagents used.
