# Supplementary material for: Pharmacological Transdifferentiation of Human Nasal Olfactory Stem Cells into Dopaminergic Neurons
Source: Stem Cells Int. 2019 May 19;2019:2945435. doi: 10.1155/2019/2945435 (PMC6545791; doi:10.1155/2019/2945435)
Supplement: Supplementary Materials — Figure S1: comparative study—stem cells versus fibroblasts—of 4 culture protocols. Figure S2: time-dependent expression of connexin 26 and consortin during OE-MSC differentiation. Figure S3: time-dependent expression of 4 dopaminergic markers during OE-MSC differentiation. Table S1: details of the 10 protocols used to differentiate OE-MSCs into dopaminergic neurons. Table S2: list of the 27 genes analyzed. Table S3: list of reagents used. [file 2945435.f1.docx]

Concise description of each supplementary figure and table

Figure S1: Analysis of gene (21) expression in human OE-MSCs and fibroblast and morphological differences between OE-MSCs and fibroblasts.

Figure S2: Time-dependent expression of connexion 26 and consortin during OE-MSC differentiation

Figure S3: Time-dependent expression of 4 dopaminergic markers during OE-MSC differentiation.

Table S1: Details of the 10 protocols used to differentiate OE-MSCs into dopaminergic neurons.

Table S2: List of the 27 genes analysed.

Table S3: list of reagents used.

# Supplementary Material

Supplementary Figures


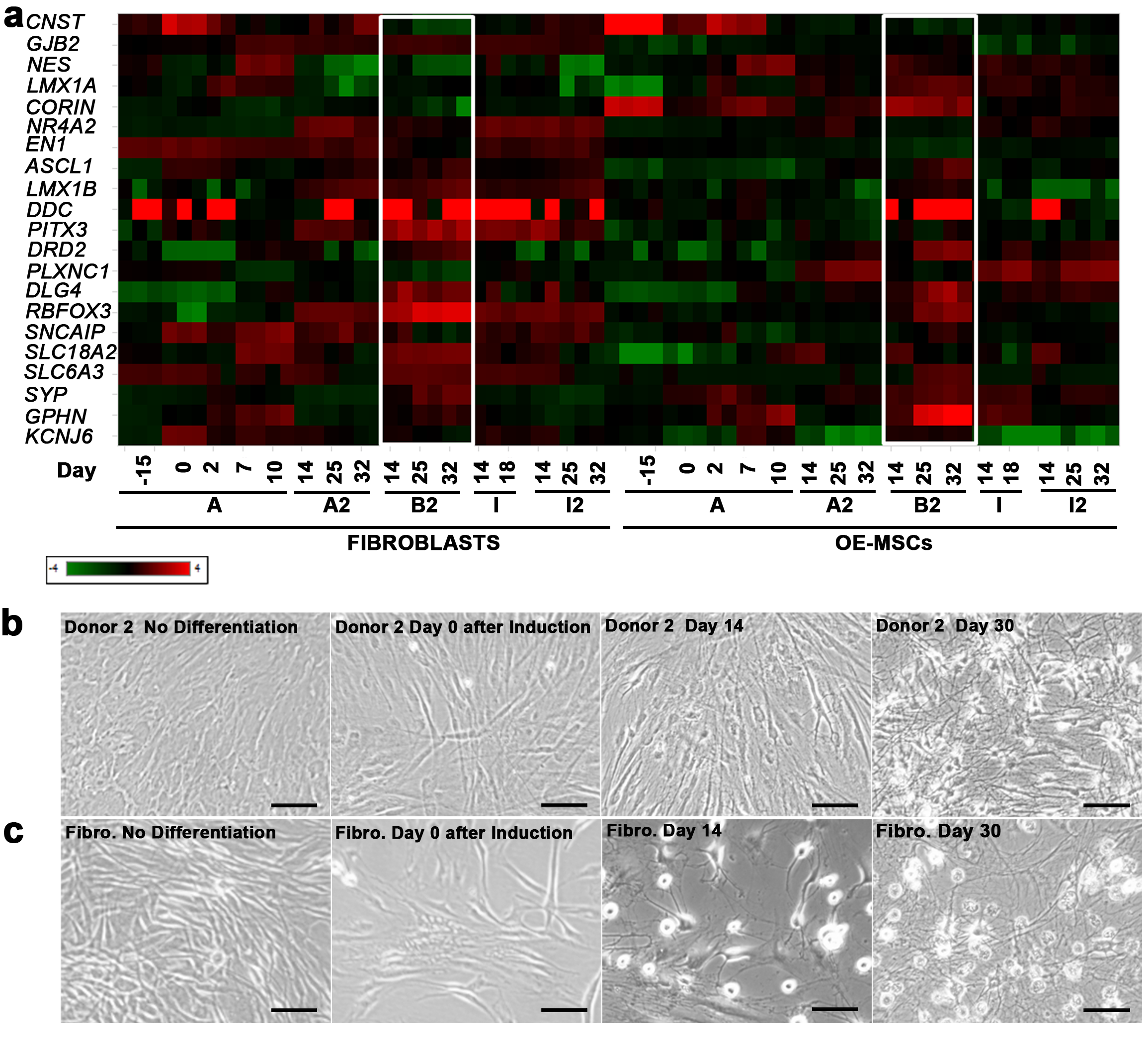


Supplementary Figure S1: Comparison of differentiation potential of OE-MSCs and human fibroblasts using 4 differentiation `protocols, A2, B2, I and I2; protocol A is used in a common first step prior to differentiation. (a) Heatmap representation of the expression of 21 selected genes: *KCNJ6*, *GPHN*, *SYP*, *SLC6A3*, *SLC18A2*, *SCNCAIP*, *RBFOX3*, *DLG4*, *PLXNC1*, *DRD2*, *PITX3*, *DDC*, *LMX1B*, *ASCL1*, *EN1*, *NR4A2*, *CORIN*, *LMX1A*, *NES*, *GJB2*, *CNST;* fibroblasts (left) and OE-MSC (right). Brightfield pictures of OE-MSCs (b) and fibroblasts (c) at 4 time points using protocol B: start of the experiment (Day -15), Day 0 after induction, differentiation Day 14, differentiation Day 30. Scale bar: 100 µm.


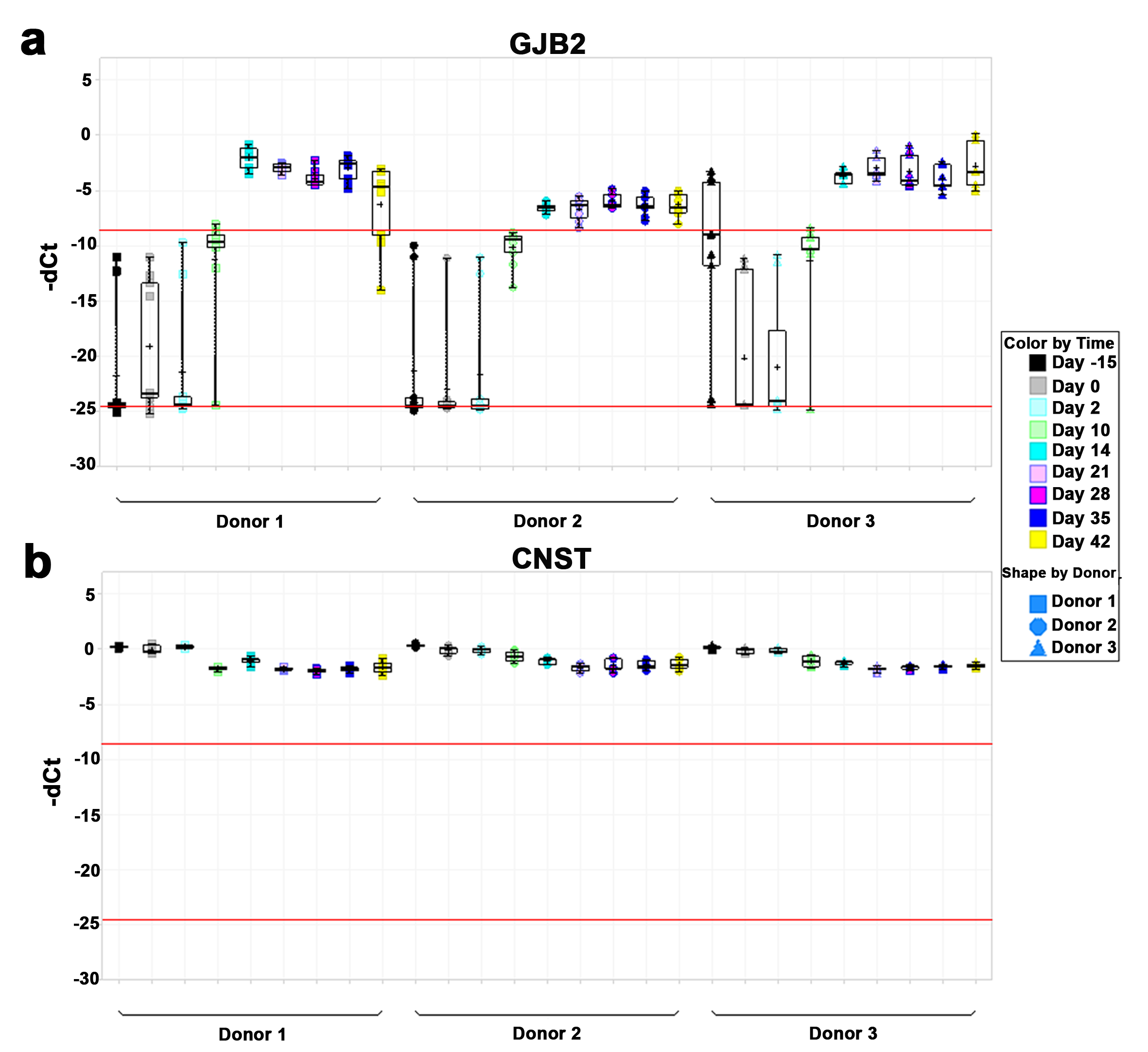


Supplementary Figure S2: Time-dependent expression of connexin 26 (GJB2) and consortin (CNST) by OE-MSCs from the 3 donors. (protocol B2). Box plot representations of the expression of *GJB2* (a) and *CNST* (b). Red lines indicate the threshold for detection (lower line) and quantification (upper line).

**
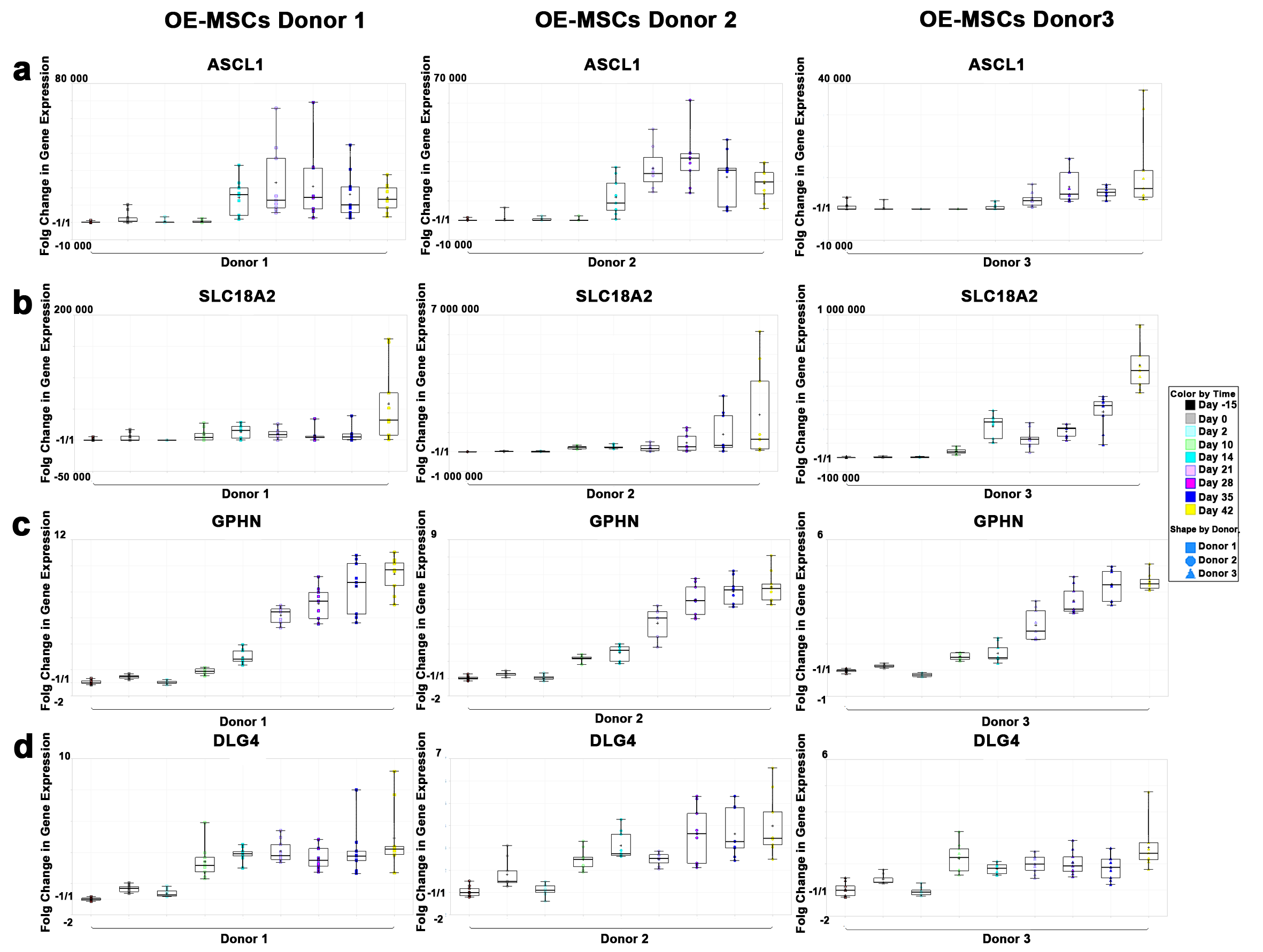
**

Supplementary Figure S3: Time- and donor-dependent expression of dopaminergic neuron-specific transcripts using protocol B2. (a-d) Box plot representations of specific dopaminergic markers (*ASCL1*, *SLC18A2, GPHN* and *DLG4*) after normalization with their basal level at Day-15 (= no treatment).

Supplementary Tables

Supplementary Table S1: Protocols tested

| **Protocols** | **Medium** |
| --- | --- |
| **Protocol A** | **Basic medium: 1:1 DMEM/F12:NB N2 0.5X (from 100X) B27 0.5X (from 50X)  + L-Glutamine P/S 1/100 + 2-Mercaptoethanol 50 µM** |
|  | 1) 15 days of induction in basic medium+FGF2+activin A. 2) PD0325901 treatment for 2 days.  3) Passage on Plo/Fibro/Lam-coated wells. Basic medium for 2days.  4) Treatment: SHH+FGF8+CHIR99021, for 3 days. 5) Treatment: SHH+FGF8+CHIR99021+purmorphamine, for 3 days.  6) Maturation medium: BDNF, GDNF, ascorbic acid, cAMP, TGFb3.  7) Day 25, maintenance medium: BDNF,GDNF, ascorbic acid, cAMP, NT3 |
| **Protocol B** | **Basic medium: 1:1 DMEM/F12:NB N2 0.5X until Day10 and then N2 1X (from 100X) B27 0.5X until Day10 and then B27 1X (from 50X)  + L-Glutamine P/S 1/100 + 2-Mercaptoethanol 50 µM** |
|  | 1) 15 days of induction in basic medium+FGF2+activin A.  2) PD0325901 treatment for 2 days. 3) Passage on Plo/Fibro/Lam-coated wells. Basic medium for 2days.  4) Treatment: SHH+FGF8+CHIR99021, for 3 days.  5) Treatment: SHH+FGF8+CHIR99021+purmorphamine, for 3 days. 6) Maturation medium: BDNF, GDNF, NT3, cAMP, CHIR99021, SB431542, noggin, LDN193189, TGFb3. 7) Day 25, maintenance medium: BDNF, GDNF, ascorbic acid, cAMP, NT3 |
| **Protocol C+A** | **Basic medium: 1:1 DMEM/F12:NB N2 0.5X (from 100X) B27 0.5X (from 50X) + L-Glutamine P/S 1/100 + 2-Mercaptoethanol 50 µM** |
|  | 1) 15 days of induction in basic medium+FGF2+activin A. 2) PD0325901 treatment, for 2 days. 3) Passage on Plo/Fibro/Lam-coated wells. Basic medium for 2days. 4) Treatment: SHH+FGF8+CHIR99021, for 3 days.  5) Treatment: SHH+FGF8+CHIR99021+purmorphamine, for 14 days. 6) Maturation medium: BDNF, GDNF, ascorbic acid, cAMP, TGFb3 7) Day 25, maintenance medium: BDNF, GDNF, ascorbic acid, cAMP, NT3 |
| **Protocol C+B** | **Basic medium: 1:1 DMEM/F12:NB N2 0.5X until Day 21 and then N2 1X (from 100X) B27 0.5X until Day 21 and then B27 1X (from 50X) + L-Glutamine P/S 1/100 + 2-Mercaptoethanol 50 µM** |
|  | 1) 15 days of induction in basic medium+FGF2+activin A. 2) PD0325901 treatment for 2 days. 3) Passage to Plo/Fibro/Lam-coated wells. Basic medium for 2days. 4) Treatment: SHH+FGF8+CHIR99021, for 3 days. 5) Treatment: SHH+FGF8+CHIR99021+purmorphamine, for 14 days.  6) Maturation medium: BDNF, GDNF, NT3, cAMP, CHIR99021, SB431542, noggin, LDN193189, TGFb3.  7) Day 25, maintenance medium: BDNF, GDNF, ascorbic acid, cAMP, NT3. |
| **Protocol D  (from Singh et al., 2017)** | **Neurobasal (NB) + B27 0.5X (from 50X) + L-Glutamine P/S 1/100** |
|  | 1) B27+ LGlutamineP/S+EGF+FGF2, for 9 Days. 2) B27+ LGlutamineP/S+EGF+FGF2+BDNF. |
| **Protocol E** | **Basic medium: 1:1 DMEM/F12:NB N2 0.5X until Day 8 and then N2 1X (from 100X) B27 0.5X until Day 8 and then B27 1X (from 50X) + L-Glutamine P/S 1/100 + 2-Mercaptoethanol 50 µM** |
|  | 1) 2 days in basic medium DMEM/F12+B27+NB+N2+BME+L-Glutamine.  2) Treatment: SHH+FGF8+CHIR99021+purmorphamine, for 6 days.  3) Maturation treatment: BDNF, GDNF, NT3, cAMP, CHIR99021, SB431542, noggin, LDN193189, TGFb3 (fibroblasts) or DAPT (OE-MSCs). |
| **Protocol A2** | **Basic medium: 1:1 DMEM/F12:NB N2 0.5X (from 100X) B27 0.5X (from 50X) + L-Glutamine P/S 1/100 + 2-Mercaptoethanol 50 µM** |
|  | 1) 15 days of induction in basic medium+FGF2+activin A. 2) PD0325901 treatment, for 2 days. 3) Passage on Plo/Fibro/Lam-coated wells. Basic medium for 2 days.  4) Treatment: SHH+FGF8+CHIR99021, for 3 days.  5) Treatment: SHH+FGF8+CHIR99021+purmorphamine, for 3 days. 6) Maturation medium: BDNF, GDNF, ascorbic acid, cAMP. 7) Day 25, maintenance medium: BDNF, GDNF, ascorbic acid, cAMP, NT3. |
| **Protocol B2** | **Basic medium: 1:1 DMEM/F12:NB N2 0.5X until Day 10 and then N2 1X (from 100X) B27 0.5X until Day 10 and then B27 1X (from 50X) + L-Glutamine P/S 1/100 + 2-Mercaptoethanol 50 µM** |
|  | 1) 15 days of induction in basic medium+FGF2+activin A. 2) PD0325901 treatment, for 2 days. 3) Passage on Plo/Fibro/Lam-coated wells. Basic medium for 2 days. 4) Treatment: SHH+FGF8+CHIR99021, for 3 days.  5) Treatment: SHH+FGF8+CHIR99021+purmorphamine, for 3 days.  6) Maturation medium: BDNF, GDNF, NT3, cAMP, CHIR99021, SB431542, noggin, LDN193189, ascorbic acid.  7) Day 25, maintenance medium: BDNF, GDNF, ascorbic acid, cAMP, NT3 |
| **Protocol I** | **Neurobasal (NB)  + B27 1X (from 50X) + L-Glutamine P/S 1/100** |
|  | 1) 15 days of induction in basic medium+FGF2+activin A.  2) PD0325901 treatment, for 2 days.  3) Passage on Plo/Fibro/Lam-coated wells. Basic medium for 2 days. 4) Treatment: SHH+FGF8+CHIR99021, for 3 days. 5) Treatment: SHH+FGF8+CHIR99021+purmorphamine, for 3 days. 6) Maturation medium: BDNF, GDNF, cAMP, ascorbic acid, DAPT. |
| **Protocol I2** | **Basic medium: 1:1 DMEM/F12:NB N2 1X (from 100X) B27 1X (from 50X) + L-Glutamine P/S 1/100 + 2-Mercaptoethanol 50 µM** |
|  | 1) 15 days of induction in basic medium+FGF2+activin A. 2) PD0325901 treatment, for 2 days. 3) Passage on Plo/Fibro/Lam-coated wells in basic medium, for 2 days.  4) Treatment: SHH+FGF8+CHIR99021, for 3 days. 5) Treatment: SHH+FGF8+CHIR99021+purmorphamine, for 3 days. 6) Maturation medium: BDNF, GDNF, cAMP, ascorbic acid, DAPT |

Summary of the 10 tested protocols to differentiate OE-MSCs into dopaminergic neurons. Basic medium is in bold

Supplementary Table S2: Main characteristics of the 27 genes assessed

| **Gene symbol** | **Alias** | **Gene Name** | **Assay ID** | **Species** |
| --- | --- | --- | --- | --- |
| *ALDH1A1* | ALDC, ALDH-E1, ALDH1, ALDH11, HEL-9, HEL-S-53e, HEL12, PUMB1, RALDH1 | Aldehyde dehydrogenase 1 family member A1 | Hs00946916_m1 | Homo sapiens |
| *ASCL1* | ASH1, HASH1, MASH1, bHLHa46 | Achaete-scute family bHLH transcription factor 1 | Hs00269932_m1 | Homo sapiens |
| *CNST* | C1orf71, PPP1R64 | Consortin, connexin sorting protein | Hs00952815_m1 | Homo sapiens |
| *CORIN* | ATC2, CRN, Lrp4, PEE5, TMPRSS10 | Corin, serine peptidase | Hs00198141_m1 | Homo sapiens |
| *DDC* | AADC | Dopa decarboxylase | Hs01105048_m1 | Homo sapiens |
| *DLG4* | PSD95, SAP-90, SAP90 | Discs large MAGUK scaffold protein 4 | Hs01555373_m1 | Homo sapiens |
| *DRD2* | D2DR, D2R | Dopamine receptor D2 | Hs00241436_m1 | Homo sapiens |
| *EN1* | _ | Engrailed homeobox 1 | Hs00154977_m1 | Homo sapiens |
| *GJB2* | CX26, DFNA3, DFNA3A, DFNB1, DFNB1A, HID, KID, NSRD1, PPK | Gap junction protein beta 2 | Hs00269615_s1 | Homo sapiens |
| *GPHN* | GEPH, GPH, GPHRYN, HKPX1, MOCODC | Gephyrin | Hs00982840_m1 | Homo sapiens |
| *KCNJ6* | BIR1, GIRK-2, GIRK2, KATP-2, KATP2, KCNJ7, KIR3.2, KPLBS, hiGIRK2 | Potassium voltage-gated channel subfamily J member 6 | Hs01040524_m1 | Homo sapiens |
| *LMX1A* | LMX1, LMX1.1 | LIM homeobox transcription factor 1 alpha | Hs00898455_m1 | Homo sapiens |
| *LMX1B* | LMX1.2, NPS1 | LIM homeobox transcription factor 1 beta | Hs01059594_m1 | Homo sapiens |
| *MAP2* | MAP2A, MAP2B, MAP2C | Microtubule associated protein 2 | Hs00258900_m1 | Homo sapiens |
| *MAPT* | DDPAC, FTDP-17, MAPTL, MSTD, MTBT1, MTBT2, PPND, PPP1R103, TAU | Microtubule associated protein tau | Hs00902193_m1 | Homo sapiens |
| *NES* | Nbla00170 | Nestin | Hs04187831_g1 | Homo sapiens |
| *NR4A2* | HZF-3, NOT, NURR1, RNR1, TINUR | Nuclear receptor subfamily 4 group A member 2 | Hs01117527_g1 | Homo sapiens |
| *PITX3* | ASMD, ASOD, CTPP4, CTRCT11, PTX3 | Paired like homeodomain 3 | Hs01013935_g1 | Homo sapiens |
| *PLXNC1* | CD232, PLXN-C1, VESPR | Plexin C1 | Hs00194968_m1 | Homo sapiens |
| *POU5F1* | Oct-3; Oct-4; OCT3; OCT4; OCT4-PG1; OCT4-pg3; Oct4-pg4; OCT4PG1; OTF-3; OTF3; OTF3C; OTF3L; OTF3P1; OTF4; POU5F1L; POU5F1P1; POU5F1P4; POU5FLC1; POU5FLC12; POU5FLC20; POU5FLC8 | POU class 5 homeobox 1 | Hs00999634_gH | Homo sapiens |
| *RBFOX3* | FOX-3, FOX3, HRNBP3, NEUN | RNA binding protein, fox-1 homolog 3 | Hs01370654_m1 | Homo sapiens |
| *SLC18A2* | SVAT, SVMT, VAT2, VMAT2 | Solute carrier family 18 member A2 | Hs00996835_m1 | Homo sapiens |
| *SLC6A3* | DAT, DAT1, PKDYS | Solute carrier family 6 member 3 | Hs00997374_m1 | Homo sapiens |
| *SNCA* | NACP, PARK1, PARK4, PD1 | Synuclein alpha | Hs01103383_m1 | Homo sapiens |
| *SNCAIP* | SYPH1, Sph1 | Synuclein alpha interacting protein | Hs00917425_g1 | Homo sapiens |
| *SYP* | MRX96, MRXSYP | Synaptophysin | Hs00300531_m1 | Homo sapiens |
| *TH* | DYT14; DYT5b; TYH | Tyrosine hydroxylase | Hs01002188_g1 | Homo sapiens |
| *TUBB3* | beta-4; CDCBM; CDCBM1; CFEOM3; CFEOM3A; FEOM3; TUBB4 | Tubulin beta 3 class III | Hs00964963_g1 | Homo sapiens |

Supplementary Table S3: Reagents used in the different differentiation protocols

| **COATING** | **Ref** | **Company** |
| --- | --- | --- |
| Poly-L- Ornithine | P3655-100 mg | Sigma-Aldrich |
| Laminin | 23017015 | Thermo Fisher Scientific |
| Fibronectin (bovine) | F1141 | Sigma-Aldrich |
|  |  |  |
| **MEDIUM** | **Ref** | **Company** |
| DMEM/F12 | 21331 | Thermo Fisher Scientific |
| Neurobasal | 21103 | Thermo Fisher Scientific |
|  |  |  |
| **SUPPLEMENT** | **Ref** | **Company** |
| N2 supplement | 17502048 | Thermo Fisher Scientific |
| B27 supplement (without Vitamin A) | 12587010 | Thermo Fisher Scientific |
| 2-mercaptoethanol | 21985-023 | Thermo Fisher Scientific |
| Fetal bovine serum (FBS) | DE14-801FH | Lonza |
| Penicillin-Streptomycin-Glutamine (100X) | 10378 | Thermo Fisher Scientific |
| Activin A | 338AC050 | Bio-Techne |
| FGF2 (Human FGF-basic) | 100-18B | PeproTech |
| PD0325901 | 4192/10 | Bio-Techne |
| SHH (Human SHH (C24II)) | 130-095-727 | Miltenyi |
| FGF8 (Recombinant Mouse FGF-8b) | 423-F8-025 | Bio-Techne |
| CHIR99021 | 4423 | Bio-Techne |
| Purmorphamine | 4551 | Bio-Techne |
| Penicillin-Streptomycin (10,000 U/mL) | 15140122 | Thermo Fisher Scientific |
| BDNF (Human/Murine/Rat BDNF) | 450-02 | PeproTech |
| GDNF (Human GDNF) | 450-10 | PeproTech |
| SB431542 | 1614 | Bio-Techne |
| Noggin (Human Noggin) | 130-103-456 | Miltenyi |
| Ascorbic Acid | A4544-25g | Sigma-Aldrich |
| cAMP (N6,2'-O-Dibutyryladenosine3':5'-Cyclic) | D0627-250 mg | Sigma-Aldrich |
| LDN193189 (12.3 mM) | SML0559 | Sigma-Aldrich |
| NT3 (Human Recombinant NT-3) | 78074 | StemCell |
| Plasmocin prophylactic | ant-mpp | InvivoGen |
